# Near-infrared photobiomodulation of blood reversibly inhibits platelet reactivity and reduces hemolysis

**DOI:** 10.1038/s41598-022-08053-y

**Published:** 2022-03-08

**Authors:** Tomasz Walski, Karolina Grzeszczuk-Kuć, Katarzyna Gałecka, Natalia Trochanowska-Pauk, Raghvendra Bohara, Albert Czerski, Konstanty Szułdrzyński, Wiesław Królikowski, Jerzy Detyna, Małgorzata Komorowska

**Affiliations:** 1 Department of Biomedical Engineering, Faculty of Fundamental Problems of Technology, Wrocław University of Science and Technology, Wrocław, Poland; 2 CÚRAM, SFI Research Centre for Medical Devices, National University of Ireland, Galway, Ireland; 3 Division of Pathophysiology, Department of Immunology, Pathophysiology and Veterinary Prevention, Wrocław University of Environmental and Life Sciences, Wrocław, Poland; 4 Department of Anaesthesiology and Intensive Care, Central Clinical Hospital of the Ministry of Interior and Administration in Warsaw, Warsaw, Poland; 5 2nd Department of Medicine, Intensive Care Unit, Medical College, Jagiellonian University, Kraków, Poland; 6 Department of Mechanics, Materials and Biomedical Engineering, Faculty of Mechanical Engineering, Wrocław University of Science and Technology, Wrocław, Poland

**Keywords:** Platelets, Biophysics, Cell biology

## Abstract

Photobiomodulation (PBM) in the red/near-infrared (R/NIR) spectral range has become widely recognized due to its anti-inflammatory and cytoprotective potential. We aimed to assess the effects of blood PBM on platelets function and hemolysis in an in vitro setting. Porcine blood samples were separated into four aliquots for this study, one of which served as a control, while the other three were subjected to three different NIR PBM dosages. The platelet count and functions and the plasma free haemoglobin and osmotic fragility of red blood cells were measured during the experiment. The control group had a considerable drop in platelet number, but the NIR exposed samples had more minimal and strictly dose-dependent alterations. These modifications were consistent with ADP and collagen-induced platelet aggregation. Furthermore, red blood cells that had received PBM were more resistant to osmotic stress and less prone to hemolysis, as seen by a slightly lower quantity of plasma free hemoglobin. Here we showed under well-controlled in vitro conditions that PBM reversibly inhibits platelet activation in a dose-dependent manner and reduces hemolysis.

## Introduction

In recent years, photobiomodulation (PBM) has attracted the interest of various scientific organizations due to studies confirming the efficacy of radiation, including in the spectral range of red light and near-infrared (R/NIR), mainly in the treatment of neurological conditions, wound recovery, pain relief, and inflammation^1–4^. The roots of PBM can be traced back to the 1960s laser production, which was primarily concerned with investigating the effects of low-energy laser radiation (hence the term low-level laser (light) therapy, LLLT) on the physiology of tissues and cells^5,6^. Although the effectiveness of noncoherent and polychromatic light sources (e.g., halogen lamps equipped with band-pass filters, light-emitting diodes) has been demonstrated many times before^7–11^, recently, the scientific community began to comment on the possibility of their practical use in medical applications, which is reflected, among others, in the reviews by Chaves et al. and Heiskanen and Hamblin^2,12^.

The question of the primary acceptor for R/NIR radiation, particularly for blood, is even more controversial^3,4,13^. Numerous studies have demonstrated the effectiveness of PBM on leukocytes, where the role of the primary photo acceptor was to be played by the protein of the mitochondrial chain, cytochrome c oxidase. However, studies conducted on erythrocytes, cells devoid of mitochondria, also showed significant effects of LLLT manifested in changes in their aggregation and deformability abilities, i.e. parameters of fundamental importance for the functions they perform in the circulatory system. Therefore, the list of potential photo acceptors is systematically expanding, currently including, among others, hemoglobin or hydrogen bonds of water molecules^4,10,14–16^.

Regardless of the molecular basis of PBMs, the effects of R/NIR light on blood in pathological states are optimistic and observed after both the use of lasers and other radiation sources, which indicates the importance of the dose as a critical parameter for achieving significant therapeutic effects such as, i.e. reducing oxidative stress and hemolysis^9,11,17^. Of the blood components, the slightest attention was paid to platelets (PLTs), which play an essential role in maintaining hemostasis. The results of the research conducted so far indicate that exposure of PLTs to the red light emitted by lasers or LEDs influences their functional activity related to the transition into an inactive form (change of shape to discoidal), suppresses sensitivity to endogenous activators and reduces the activity of enzymes in the arachidonic acid cascade. The above effects of blood PBM were observed both in in vitro and in vivo models^18,19^.

Because of the recently emerging preclinical trials of medical applications aimed at exposing blood to R/NIR radiation both ex vivo and in vivo (e.g., during extracorporeal circulation)^17,20,21^, for the prevention of PLT apoptosis induced by various insults, and thus an extension of PLT lifespan, we provide in this paper for the first time studies showing the time-reversible inhibition of PLT activity due to NIR in the pig blood PBM depending on the radiation dose used. Moreover, blood PBM was also beneficial for red blood cells (RBCs) as it helped reduce their breakdown in vitro.

The present manuscript describes the applied assay protocols and an experimental setup for blood PBM, enabling precise control of the PBM dose and blood temperature. Experiments to test different doses of PBM on PLTs activation, reversibility of induced processes, and the level of RBCs destruction are presented in the results section. Finally, the discussion section summarizes our results, compares them with existing research, and provides a mechanistic interpretation, future directions and applications.

## Materials and methods

### Research material

The research material was venous blood from Polish Landrace pigs, sampled survival for 3.8% trisodium citrate (POCH, Gliwice, Poland), in 1 part of the reagent to 9 parts of blood, into sterile polypropylene tubes (FL Medical, Torreglia, Italy). Measurements were started no later than 1 h after blood collection.

### Experimental setup for blood PBM

**Figure 1:**
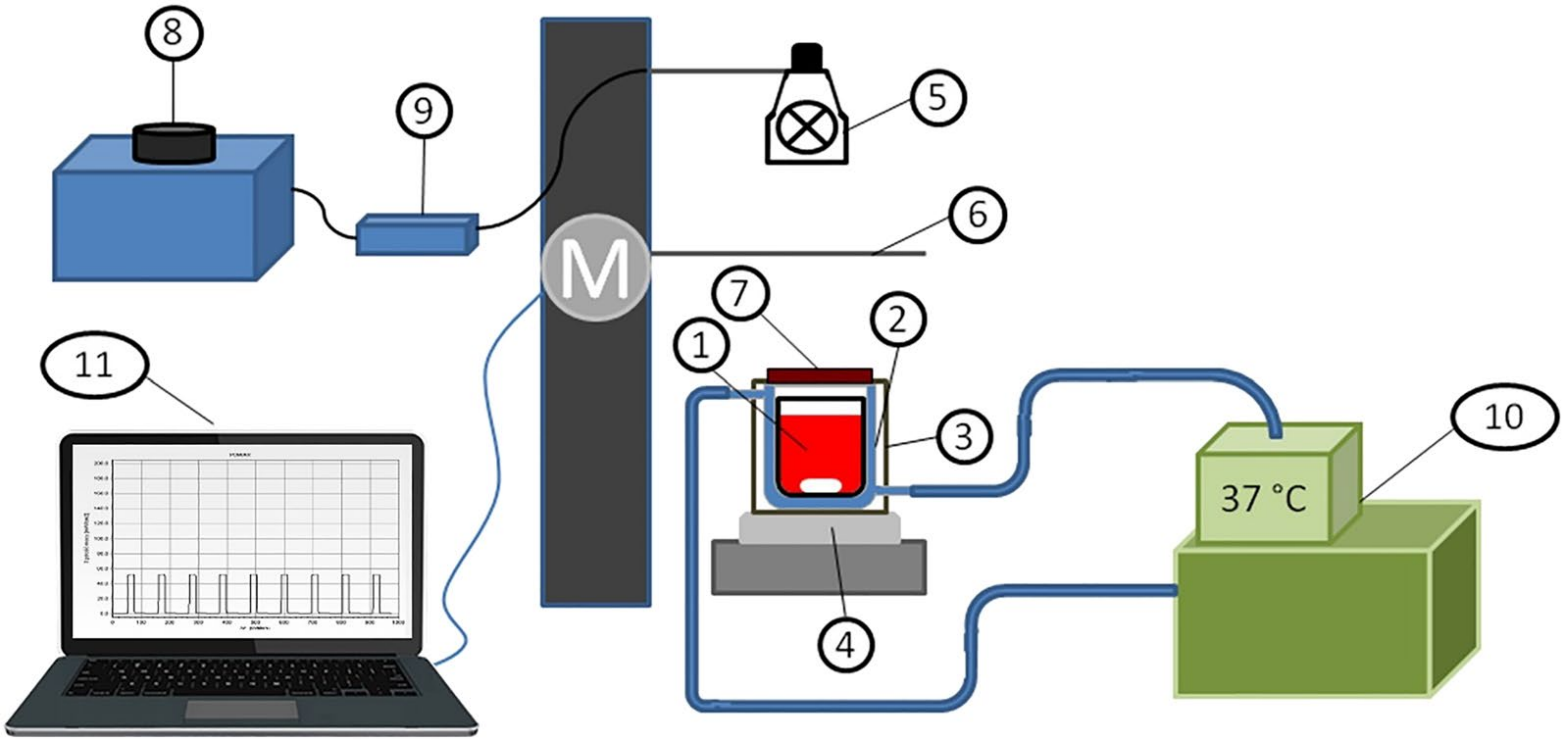
Diagram of the system for PBM of blood with NIR radiation, where: 1—blood with a magnetic bar are placed in a polypropylene container, 2—water-jacketed glass chamber, 3—external housing, 4—the electromagnetic stirrer, 5—halogen reflector, 6—moving shutter, 7—NIR filter, 8—autotransformer, 9—switched-mode power supply, 10—thermostat, 11—a computer with an application controlling the operation of the motor ‘M’ responsible for opening the shutter during pulsed exposure.

**Figure 2:**
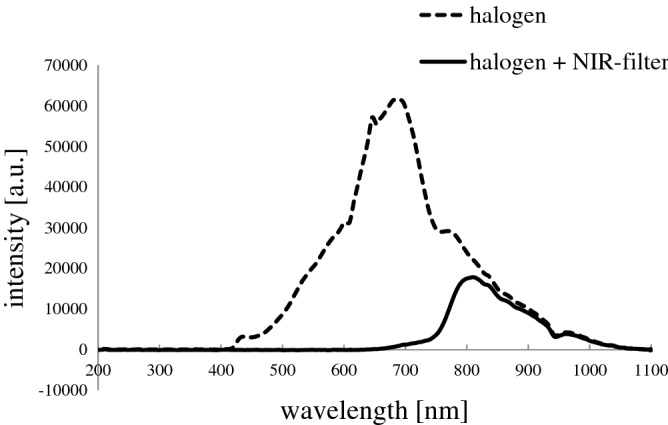
The applied system’s spectrum for blood PBM consists of a halogen reflector and a broadband NIR filter. The transmission of electromagnetic radiation in this system starts with a rising slope, which begins at approx. 700 nm. Then, two bands can be distinguished—the first, wide, with a maximum intensity of 800 nm, and the second, of low intensity, with a maximum of 960 nm. The radiation intensity consisted of 86% of the NIR spectral range waves, while 14% were electromagnetic waves from the red light range.

The scheme of the test stand is shown in Fig. 1. A polypropylene chamber containing a 10 ml blood sample was placed in a water-jacketed glass chamber fitted to its dimensions. The external circulation of the water flowing through the jacket forced by the circulation pump of the thermostat (E5 thermostat with water bath B12, Medingen, Germany) allowed maintaining a constant sample temperature at (37.0 ± 0.2) °C. The chamber was closed in a housing with a NIR filter opening. It was placed on a motorless, electromagnetic stirrer (ES 21H, WIGO, Poland), which allowed blood stirring rotational speed equal to 250 rpm using cylindrical, magnetic bar (Rotilabo 0.973, Carl Roth GmbH + Co. KG Karlsruhe, Germany) covered with polytetrafluoroethylene. The NIR irradiator consisted of a halogen reflector (MR11 + C GU4 30°, 10 W, 12 V, ANS Lighting, Warsaw, Poland), which was placed on a tripod above the near-infrared filter (NIR IFG 098, Schneider-Kreuznach, Bad Kreuznach, Germany). The samples were exposed to photons with a wavelength range (750–1100) nm (Fig. 2). The irradiance reaching the sample’s surface was regulated by changing the voltage on the autotransformer (PowerLab VT5-1, Guangdong, China). The voltage converter (Indel YT150, Warsaw, Poland) was connected, powering the halogen reflector. NIR radiation pulses were obtained by crossing the optical path: a halogen reflector—NIR filter through a shutter, the movement of which was controlled by a developed computer application. The blood exposure system to NIR radiation consisted of four identical measuring stations, and the standard part was a thermostat with a hydraulic system of parallel-connected, water-jacketed chambers. The temperature of the samples did not differ by more than 0.1 °C. NIR radiation did not change the temperature of the samples.

### Computational fluid dynamics (CFD) analysis

**Figure 3:**
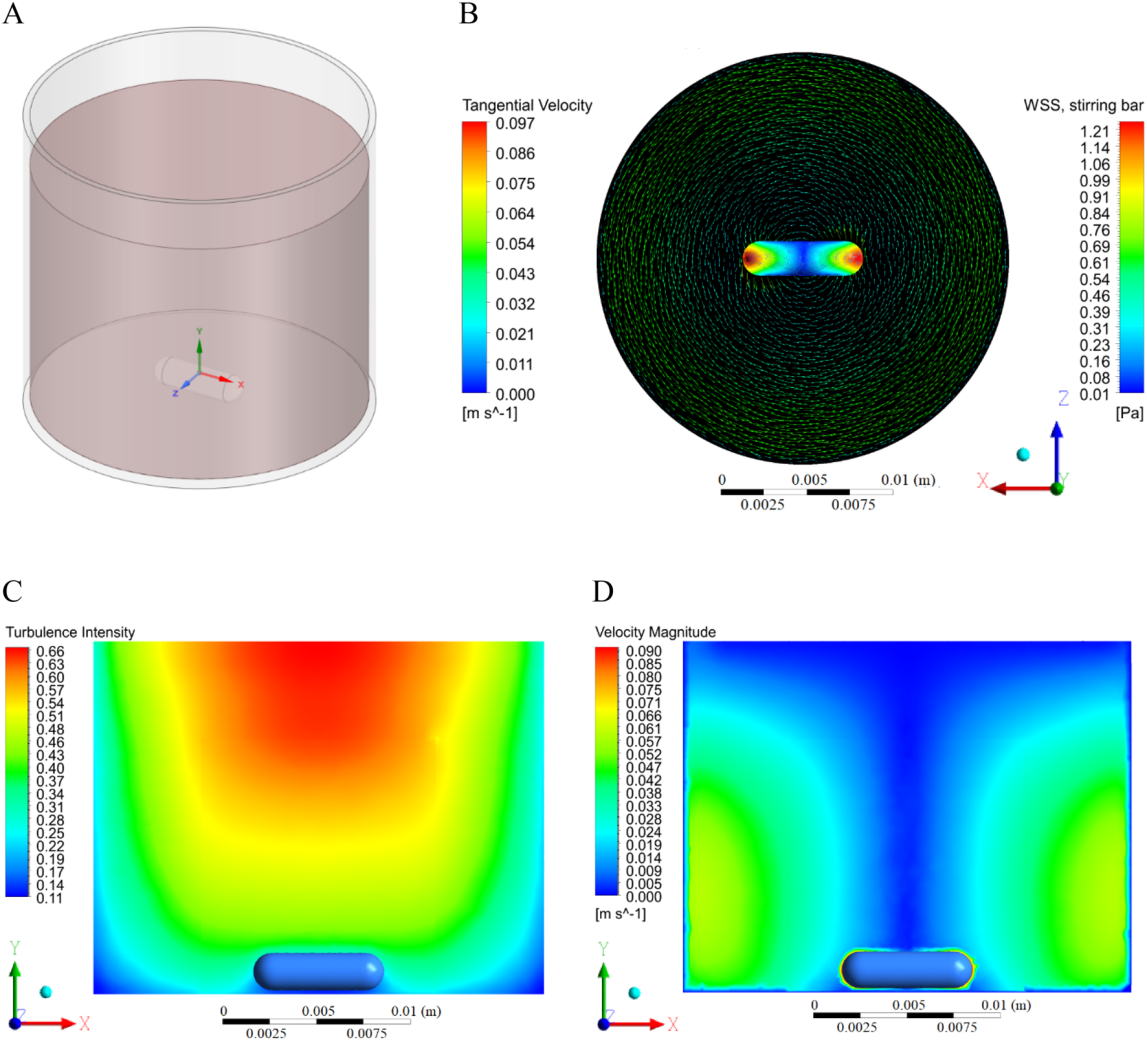
3D model of the stirring chamber with blood and magnetic bar (**A**). Tangential velocity of the stirred blood and wall shear stress (WSS) contours distribution on the stirring bar surface in the horizontal plane (**B**). Turbulence intensity (**C**) and velocity magnitude profile (**D**) within the chamber during the stirring process (vertical plane).

This study involved a sliding mesh modelling approach described by Cortada-Garcia et al.^22^ with the mesh build of 61,590 nodes and 313,507 elements. The CFD method was applied to estimate and visualise the wall shear stress and velocity profiles of blood in the mechanically stirred chamber (Fig. 3). Blood density was set for 1060 kg/m^3^, blood viscosity for 0.0035 Pa s. The temperature was constant and equal to 37 ℃. The boundary conditions for zero slip velocity at rigid walls were employed in the simulations. The magnetic bar was rotating at 250 rpm, corresponding to 26 rad/s. All the processes, including model design, meshing, and simulations, were performed using Ansys Fluent in ANSYS Workbench 20.2.

### Measurement procedure and dosage of NIR radiation

The blood sample was introduced to the measuring system and heated for 15 min, stabilizing its temperature at 37 °C. The level of the initial values of the parameters included in the assay panel was determined. Then, the blood irradiation procedure was started with 1-s NIR radiation pulses with irradiance in the range (0.75–50) mW/cm^2^ (Optel radiometer, Opole, Poland) repeated every 150 s for 8 h. The dose-ranging study was performed in two groups: (GP1) 0, 0.75, 1.5, 7.5 mW/cm^2^; (GP2) 0, 1.5, 15, 50 mW/cm^2^. The irradiance for which the best results were obtained was selected for detailed analyzes. Samples for PLT determinations were taken every 2 h. In contrast, collagen and adenosine diphosphate (ADP) induced aggregation and degree of hemolysis after 4 and 8 h. Hemolysis curves were performed after 4 h of the experiment when there was no difference in the level of hemolysis. In the second series of experiments, blood was exposed to NIR radiation with an irradiance of 1.5 mW/cm^2^ with an entire pulse sequence, which was applied for 2, 4, or 8 h.

### Determination of discoid platelet number by using the direct method

The determination of the change in the number of discoid PLTs was performed by the direct counting method. An optical microscope (Nikon Eclipse E50i, Tokyo, Japan), a Bürker chamber, and a Thrombo Plus kit (Sarstedt AG & Co. KG, Nümbrecht, Germany) were used for this purpose. Quality control of the technique used to assess PLT number included diameter determination at different experiment stages for 100 cells at each time point (Supplementary Fig. 1) and additional study using antiplatelet aggregating agent—acetylsalicylic acid (Supplementary Figs. 2, 3). Typically, the distribution of PLTs was gaussian in shape with a mean value of the cell diameter equal to 2.7 μm, ranging from 1.9 to 3.7 μm (Supplementary Table 1). The same trained person performed measurements using NIS elements D software (Nikon, Tokyo, Japan).

### PLT aggregation test

Measurements were made on a dual-channel Lumi-aggregometer operating in impedance mode (model 700, Chrono-log, USA) as previously described^20^. Briefly, 450 µl of either blood and PBS were pipetted into a plastic cuvette (#367, Chrono-log, Havertown, USA) containing a measuring electrode and a magnetic stir bar (#370, Chrono-log, Havertown, USA) and rotated at a speed of 1100 rpm. The sample was incubated for 5 min at 37 °C, the baseline was set, and the agonist was added: 10 µl ADP 1 mM (#384, Chrono-log, Havertown, USA) and 50 µl CaCl_2_ 3 mM (POCH, Gliwice, Poland) or 2 µl collagen (#385, Chrono-log, Havertown, USA). The measurement lasted 6 min from the addition of the agonist. Impedance changes were recorded using the Aggro/link8 program.

### Osmotic fragility and hemolysis curve

The measurement of the osmotic fragility of blood and the analysis of the hemolysis curve were performed according to the previously described methodology^23^. Briefly, to each of a series of test tubes containing 1 ml of NaCl solution in the concentration range (0–145) mM NaCl buffered in 10 mM PBS, pH 7.4, 10 µl of blood was added, mixed (IKA TTS 3, IKA-Werke GmbH & Co KG, Staufen, Germany) and incubated for 30 min. The samples were then centrifuged at 1750×*g* for 4 min (MPW 350 centrifuge, MPW Med. Instruments, Warsaw, Poland). The level of free hemoglobin in the obtained supernatant was determined spectrophotometrically (Nicolet Evolution 60, Thermo Scientific, Waltham, USA) by measuring the absorbance at 540 nm against distilled water. The hemolysis curve was performed twice, the first time initially estimating the osmotic fragility value and the second time with high precision and resolution, especially in the range of NaCl concentrations, where a sharp increase in absorbance was observed. The value of osmotic fragility is the concentration of NaCl at which half of the blood cells were hemolyzed. The straight-line slope approximating the transition area of the hemolysis curve was taken as the measure of the dispersion of individual osmotic fragility within the population.

### Hemolysis study

The concentration of free hemoglobin in plasma (PFHgb) was determined by the Drabkin method as previously described^17^. Briefly, plasma, obtained by centrifugation of citrated blood for 10 min at 1750×*g*, was mixed with Drabkin’s reagent (Aqua-Med, Łódź, Poland) in sample to diluent volume ratio 1:10 (dilution factor of 11), and incubated for 5 min. Finally, the absorbance measurement using semi-micro cuvettes with a light path length of 1 cm (Sarstedt, Nümbrecht, Germany) at 540 nm was performed against distilled water, and each sample was measured in triplicate.

### Statistical analysis

The analysis of the research results and the figures were performed using statistical package Statistica 12.0 (StatSoft Inc./TIBCO Software, Palo Alto, USA). After determining the basic statistical measures, the hypothesis of the normality of the empirical distribution was verified using the Kołmogorow–Smirnow (K–S) and Shapiro–Wilk (S–W) tests. The normality of the distribution of variables was also graphically assessed using histograms and standard probability plots of variables.

In the case, where at the significance level p = 0.05, the null hypothesis H0: the distribution of the analyzed variable is a normal distribution, was accepted, parametric tests, t-test, or MANOVA multivariate analysis of variance for repeated measures were performed. After verification of the assumptions of variance analysis and rejecting the null hypothesis about the lack of differences in the compared populations, Tukey’s post hoc tests were performed.

Nonparametric tests were used when the S–W and/or K–S tests indicated rejection of the hypothesis of normal distribution. Comparisons of pairs of dependent variables were performed with the Wilcoxon pair test. However, in the analysis of variance for repeated measurements, the Friedman ANOVA test was used, followed by Dunn’s post hoc test in case the hypothesis about the lack of differences between the studied samples was rejected. Differences were considered statistically significant for p < 0.05.

### Bioethical issues

A local II Ethical Review Board in Wrocław (II Lokalna Komisja Etyczna do Spraw Doświadczeń na Zwierzętach Uniwersytetu Przyrodniczego we Wrocławiu) approved the study protocol (approval No. 71/2013). According to the European Directive 2010/63/EU, each animal was provided humane conditions and care to protect animals used for scientific purposes.

## Results

### Comutational fluid dynamics simulations

The CFD findings suggest working with the semi-laminar flow (Reynolds Number not higher than 2900). The highest level of wall shear stress was observed on the stirring bar endings and outer wall of the chamber, which were 1.2 Pa and 0.7 Pa, respectively (Fig. 3B). Turbulence intensity is shown in Fig. 3C. The values are lower than 1%, so it is considered low. The stirring process within the cylindrical cuvette leads to the propagation of the tornado effect, which was confirmed by the velocity profile in Fig. 3D.

### PBM efficiently inhibits PLTs activation in a dose-dependent manner

**Figure 4:**
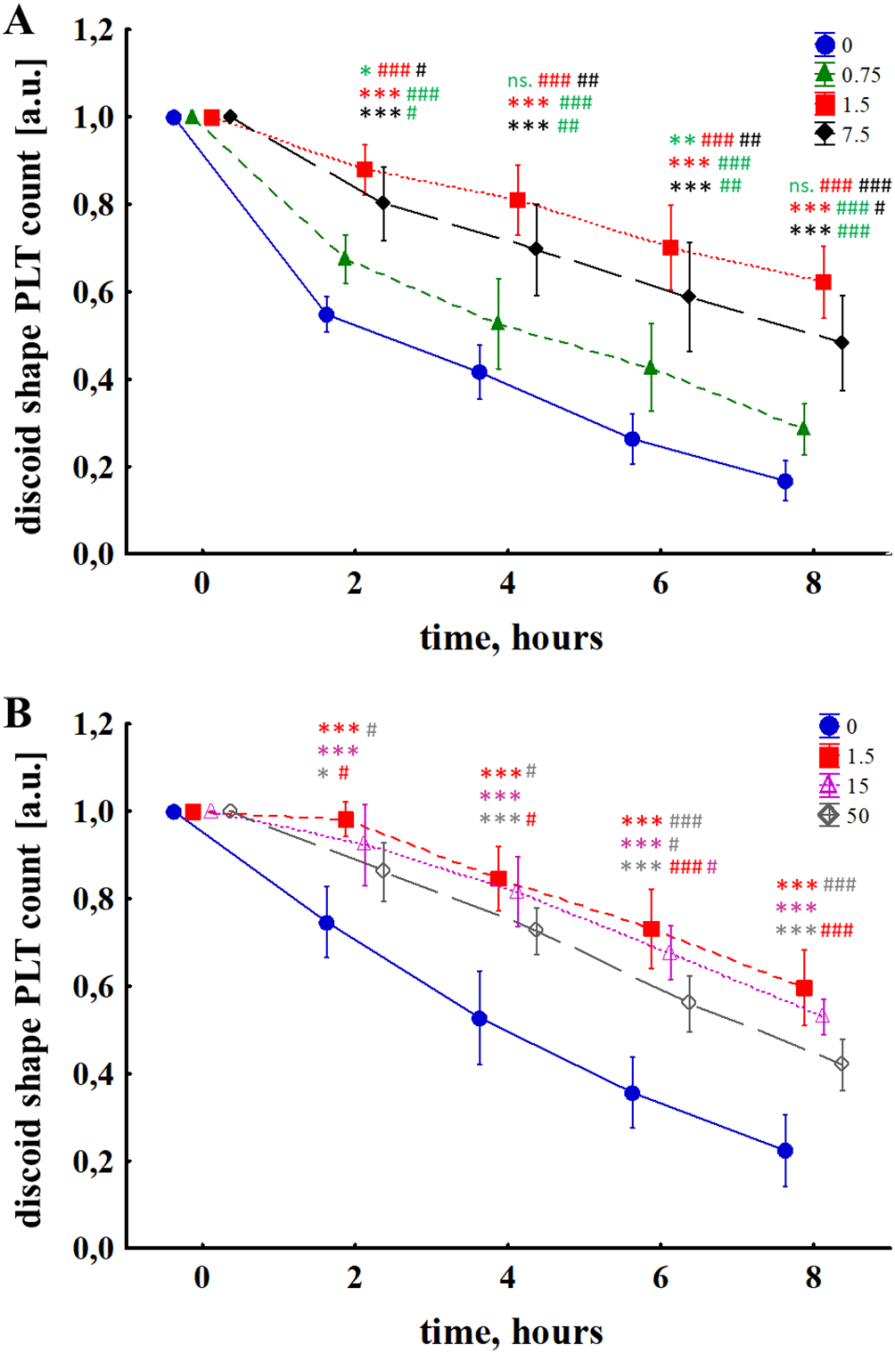
Change in the number of discoid shape PLT subjected to NIR PBM with irradiances: (**A**) 0, 0.75, 1.5, 7.5 mW/cm^2^; (**B**) 0, 1.5, 15, 50 mW/cm^2^. The MANOVA results for interaction, time, and group were p < 0.001 in each case. The results are presented as mean values ± standard deviation. The number of measurements n = 5 for each group of measurements. The asterisk (*) indicates the differences with regard to the CTR group and the hash (#) indicates the differences between the individual NIR doses at particular time points. *p < 0.05, **p < 0.01, ***p < 0.001; ^#^p < 0.05, ^##^p < 0.01, ^###^p < 0.001.

In the experimental model used, along with the incubation time, the PLTs are gradually activated, as evidenced by the decrease in the number of discoid shape cells in each group (p < 0.001) (Fig. 4A). The most significant reduction in PLT was observed during the first 2 h of incubation—35% in the control (CTR) group (0 mW/cm^2^), were at the end of the experiment, less than 20% of PLTs remained. The effect of the lowest NIR dose, which corresponded to an irradiance of 0.75 mW/cm^2^, was modest, as evidenced by a marginally higher PLT count at the two measurement points (t (2,6 h); p < 0.05, p < 0.01, respectively) when compared to CTR. PLT activation was significantly inhibited by increasing the irradiance value to 1.5 mW/cm^2^ (p < 0.001 vs CTR at time points t (2–8 h)). So, in general, 0.75 mW/cm^2^ was weaker when compared to higher NIR doses tested but marginally better than CTR. In the middle of the experiment, the PLT count was over 82% of the initial PLT value, and at the end of the investigation, the NIR 1.5 mW/cm^2^ test had three times more PLTs—61%—than the CTR (p < 0.001). There is also no doubt that the number of PLTs in the 1.5 mW/cm^2^ test is significantly greater than in the 0.75 mW/cm^2^ group (p < 0.001 at each time point). Further dose escalation, corresponding to the irradiance of 7.5 mW/cm^2^ and 15 and 50 mW/cm^2^ (Fig. 4B), proved effective in inhibiting PLT activation as evidenced by the more significant number of discoid-shaped PLTs at individual time points compared to the CTR group. The highest effectiveness of NIR blood PBM was noted for the 1.5 mW/cm^2^ group, for which, unlike the others, PLT was higher than in 50 mW/cm^2^ (p < 0.05 at t (2–4) and p < 0.001 at t (6–8)). Therefore, in further experiments, the effects of NIR blood PBM with an irradiance of 1.5 mW/cm^2^ were analyzed in detail. PBM parameters were summarized in Table 1.

**Table 1:** Photobiomodulation parameters—irradiance dependent dosing of NIR.

Irradiance (mW/cm^2^)	0.75	1.5	7.5	15	50
Power (mW)	3.39	6.78	33.9	67.8	226
Continuous-wave mode	no	no	no	no	no
Pulse sequence	1 s of NIR–150 s of break				
Duration of treatment (h)	8	8	8	8	8
Irradiation time (s)	192	192	192	192	192
Spot area (cm^2^)	4.52	4.52	4.52	4.52	4.52
Energy (J)	0.65	1.3	6.5	13	43
Fluence (J/cm^2^)	0.14	0.29	1.44	2.9	9.6

**Figure 5:**
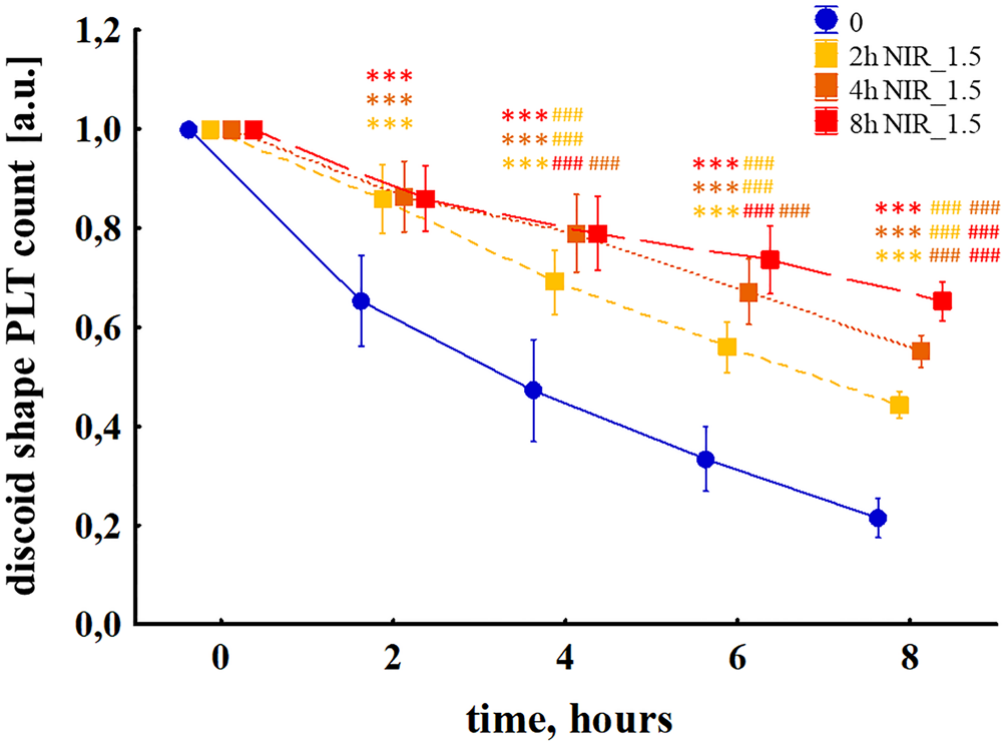
Change in the discoid shape PLT number subjected to NIR PBM with an irradiance of 1.5 mW/cm^2^ (NIR_1.5) applied for 2, 4, or 8 h. The MANOVA results for interaction, time and group were p < 0.001 in each case. The results are presented as mean values ± standard deviation. The asterisk (*) indicates the differences concerning the CTR group, and the hash (#) indicates the differences between the individual NIR doses at particular time points. The number of measurements n = 11. ***p < 0.001, ^###^p < 0.001.

Discontinuation of the blood PBM was followed by cessation of the light inhibitory effect on PLT activity. Figure 5 shows the changes in discoid shape PLT in the case of blood exposure to PBM with an irradiance of 1.5 mW/cm^2^ for 2, 4, or 8 h. Already 2 h after the end of the disclosure, i.e., at t (4 h), there is a significant difference in PLT between the 2 h of NIR 1.5 mW/cm^2^ sample and the other two—4 h and 8 h—which were still subject to PBM (69% vs 79% and 79%, p < 0.001 for both comparisons, respectively). A similar phenomenon was observed when PBM was stopped after 4 h. The difference between PLT number in samples 4 h of NIR 1.5 mW/cm^2^ and 8 h of NIR 1.5 mW/cm^2^ at t (6 h) was 6% (ns) and increased to 10% (p < 0.001) at t (8 h), which indicates faster activation PLTs after completion of PBM. PBM parameters were summarized in Table 2.

**Table 2:** Photobiomodulation parameters—time-dependent dosing of NIR.

Irradiance (mW/cm^2^)	1.5	1.5	1.5
Power (mW)	6.78	6.78	6.78
Continuous-wave mode	no	no	no
Pulse sequence	1 s of NIR–150 s of break		
Duration of treatment (h)	2	4	8
Irradiation time (s)	48	96	192
Spot area (cm^2^)	4.52	4.52	4.52
Energy (J)	0.3	0.7	1.3
Fluence (J/cm^2^)	0.07	0.14	0.29

### Platelet function testing reveals decreased aggregation following PBM

**Figure 6:**
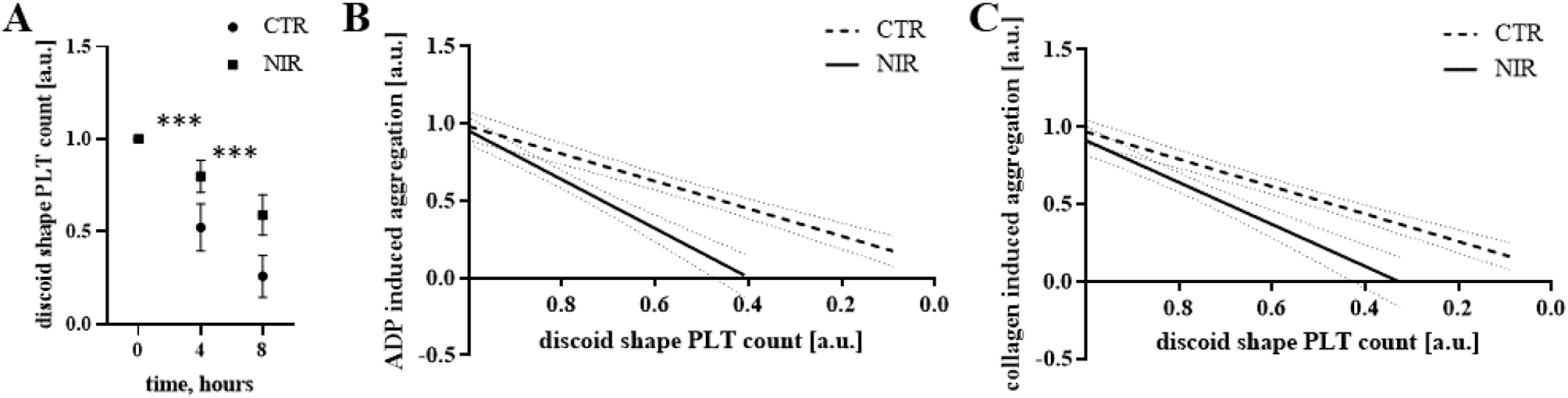
PLT function tests related to discoid shape PLT count changes during the experimental procedure. (**A**) A significant decrease in the number of discoid shape PLT in both groups (p < 0.001), while in NIR_1.5 PBM samples (filled square) the observed loss is characterized by a lower value throughout the experiment than for the CTR samples (filled circle). Collagen (**B**) and ADP (**C**) induced aggregation versus PLT for CTR (bold dashed line) and NIR_1.5 (bold solid line) groups approximated by linear regression. The slope coefficients in the two groups were significantly different (p < 0.01 for collagen and p < 0.001 for ADP). The thin dashed line means 95% confidence interval. The results are presented as mean values ± standard deviation. ***p < 0.001 at a given measuring point. The number of measurements n = 20.

Whole blood aggregometry with collagen and ADP as agonists indicated a more significant approx. 30% and 20%, respectively, the reactivity of NIR radiation-modified PLTs with an irradiance of 1.5 mW/cm^2^ (Table 3). However, considering that PBM samples also significantly differed in the number of PLTs at individual experiment stages compared to CTR samples, the data were subjected to regression analysis (Fig. 6). The slope coefficients of the regression line illustrating the dependence of both ADP and collagen-induced aggregation on the PLT count, in the case of the CTR group, were respectively (0.8877 ± 0.0841) and (0.8870 ± 0.0728), which indicates that the percentage reduction in the number of PLTs in the sample corresponded to decrease in aggregation with similar percentage intensity. Blood exposure to NIR radiation sequences resulted in a significant increase in the values of the slope of the regression line—for ADP (1.583 ± 0.1611) (p = 0.0002 against the CTR test) and collagen (1.359 ± 0.1620) (p = 0.0058 against the CTR test). This most likely indicates that some PLTs did not respond to agonist activity or their reactivity was impaired by 33% in the case of ADP and 40% in collagen.

**Table 3:** Changes in ADP and collagen-induced PLT aggregation.

Agonist	Time (h)	CTR			NIR			p-value
		Percentile 25	Median	Percentile 75	Percentile 25	Median	Percentile 75	
ADP	4	0.09	0.27	0.43	0.50	0.59	0.78	0.0005
	8	0.14	0.20	0.33	0.31	0.50	0.77	0.0011
Collagen	4	0.43	0.48	0.58	0.51	0.63	0.76	0.012
	8	0.18	0.25	0.31	0.19	0.29	0.48	ns

Aggregation results were compared with the Wilcoxon pair test. The number of measurements n = 20.

### Hemolysis parameters served as an indicator of the integrity of the erythrocyte cell membrane

**Figure 7:**
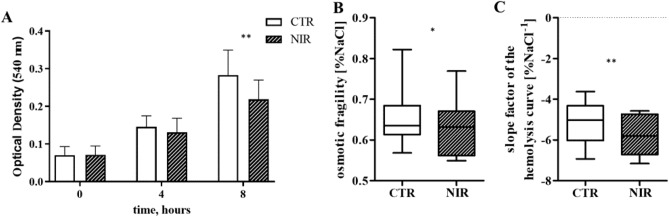
Changes in OD at 540 nm (ordinate) represent the results obtained from the plasma-free hemoglobin spectrophotometric assay that indicated erythrocyte breakdown with time of blood incubation in the experimental system (MANOVA test results for interaction, time, and group were p < 0.001, p < 0.001, p < 0.05, respectively) (**A**). The results are presented as mean values ± standard deviation. **p < 0.01 for CTR (empty bar) vs NIR 1.5 mW/cm^2^ (diagonal pattern) at a given time point. Furthermore, significant changes (***p < 0.001) were also found compared to other time points within each group, and different time points for intergroup comparison. The number of measurements n = 20. Differences in the parameters of the hemolysis curve at t (4 h): osmotic fragility (**B**) and the slope of the hemolysis curve (**C**) (Wilcoxon’s test in both cases). The results are presented as the median value and the interquartile range. The number of measurements n = 10.

Changes in hemolysis during the experiment served as an indicator of the integrity of the erythrocyte cell membrane. With the incubation time, RBCs were broken down (p < 0.001) (Fig. 7A). There was no significant difference in hemolysis between the groups after the first 4 h of incubation; however, RBC degradation proceeded throughout the experiment (p < 0.001). The hemolysis rate expressed as optical density measured at 540 nm in the CTR group was 0.283 ± 0.66, while 0.219 ± 0.51 in the NIR sample (p < 0.01). The analysis of the hemolysis curves at t (4 h) was also performed. The differences in osmotic fragility indicate that the cells subjected to NIR PBM were characterized by more excellent resistance to osmotic stress (p < 0.05), and the difference in the slope of the hemolysis curve indicates a narrower distribution of the population in terms of osmotic properties (p < 0.01) (Fig. 7).

## Discussion

PLTs are small anucleate fragments of megakaryocytes whose primary function is to form a hemostatic plug to restore continuity of damaged blood vessels^24^. Their relatively simple structure means that they can be an attractive model system for studying the effect of light on a cell. However, PLTs are extremely sensitive to stimuli such as e.g. calcium ions, agonists (e.g. thrombin, collagen, ADP, or thromboxane A_2_ (TxA_2_)), and shear stresses^24,25^. PLT activation is associated with a cell shape change, PLT aggregation, and the release of PLT constituents, affecting hemostasis and rheological properties of blood.

Blood cells damage can occur when exposed to forces not typically seen in the body, which is a common problem of mechanical circulatory support devices such as, e.g. blood pumps used in extracorporeal circulation systems, ventricular assist devices, the total artificial heart, and prosthetic heart valves. High shear stress is one of the most important factors to cause hemolysis and affect PLT activation and aggregation^26^. This independent activation agonists process is related to the magnitude of shear stress acting on a cell and the cell’s exposure time to that shear stress^27,28^. The CFD simulation was performed to determine flow conditions during the experiment. The results showed that the flow inside the cuvette was semi-laminar with low turbulence intensity. Wall shear stress values were comparable to physiological ones; mainly, similar wall shear stress occurs in the human common carotid artery^29^. However, it should be taken into account that only the cells that pass near a solid surface are subjected to maximum stress during the flow in the blood vessels. In our chamber, cells were constantly exposed to lower shear stress when compared with those occurring in, e.g. VAD. Still, due to stress accumulation within a relatively long time of incubation, PLT activation is observed, followed by secretion of their hemostatic-activating products and finally, PLT depletion, which has been shown^30^.

Our previous studies showed the limitation of PLT loss and PLT function stabilization during extracorporeal circulation in an animal model of cardiopulmonary bypass^20^. Using a relatively simple in vitro model where blood cells were exposed to mechanical stress due to magnetic bar stirring, we tested the effects of different PBM doses on PLT activation and function. Our results revealed for the first time that polychromatic NIR radiation inhibits PLTs in a dose-dependent manner, which was shown by the changes in the number of discoid-shape cells at the time of the experiment (Figs. 4, 5, 6A). The irradiated cells had limited reactivity to both ADP and collagen agonists. Contrary to our results, Irmak et al. recently used polychromatic light range from 600 to 1200 nm for PRP irradiation during storage and showed opposite effects measured in increased growth factors, ATP secretion, and calcium release from PLTs. Light exposed PLTs were characterized by extended lamellipodia, numerous filopodia, and PLT agglomeration. P-selectin expression was significantly increased^31^.

In Irmak et al., energetic parameters of delivered radiation (radiant fluence and irradiance) were higher than in our study. However, a part of the irradiance range overlaps and could be considered similar values. The main difference could be seen when comparing values of total energy delivered to the sample, which was tremendous. In our study, the range was 0.016–4.3 J/cm^3^, while in Irmak et al., the corresponding values were equal to 54–540 J/cm^3^ (PRP volume of 0.1 ml exposed to radiation). Another critical difference is the sample composition. Irmak et al. exposed PRP, while in our study WB was used. With this in mind, the difference between the doses applied in both works will turn out to be even more significant due to NIR absorption by hemoglobin in erythrocytes. Assuming that the shape of the emission spectra of both light sources were similar (which is not obvious and could also be the reason for conflicting results), we can state that NIR irradiance’s effect on PLT is biphasic nature. The two-phase, dose-dependent response of the biological system to the action of low-power laser radiation was described in detail by Huang et al.^32^.

Other studies aimed at assessing the effect of red to NIR radiation on PLTs were carried out using in vitro, in vivo, and ex vivo techniques. It is well established that red light, generated by He–Ne lasers (632.8 nm) and LED sources, regardless of the research framework used, influence the PLT-mediated coagulation system, lowering the aggregation induced by: ADP, collagen, epinephrine, ristocetin, PLT activation factor (PAF), fibrinogen, adrenaline, ristomycin, and the thrombin receptor activating peptide (TRAP)^18,19,33–37^. The results of thrombelastography showed a decrease in the rate of thromboplastin and thrombin formation, which inhibits the conversion of fibrinogen to fibrin. Thus, blood clotting is reduced and the clot formation time is extended; the hypercoagulation syndrome is formed^33^. At the same time, no change in the total PLT count was observed^34,35,38^, but there has been a redistribution of the population’s shape. The reduction in spherical (activated) cells was accompanied by increased discoidal (non-activated) cells. This proves a decrease in the degree of their activation in the population of PLTs exposed to red light^34,35^.

In Brill et al.^18,38^, irradiation of blood inhibited the settling of PLTs on the surface under conditions simulating the flow in blood vessels. This effect was dose-dependent (time-regulated) and finally reversible after 30–60 min from the end of PBM. PLT activation is accompanied by a change in GPIIb-IIIa receptor conformation and the expression of alpha-granular (P-selectin) content on the PLT surface. Whole blood irradiation leads to inhibition of fibrinogen binding and P-selectin expression after activation by TRAP^18,38^. The phenomenon occurs for relatively low concentrations of the agonist used (6.25 µM). At higher concentrations (in the order of 25 μM), the effect is negligible. In the first case, PLT reversible dysfunction was noted—inhibition lasted 15 min, and fibrinogen rebinding occurred after 30 min^18^. In Spasov et al.^34^, the sensitivity of irradiated PLTs to TxA2 and indirectly to the activities of COX and thromboxane synthetase (TxS) were determined. Collagen-activated PLTs were added to unmodified or irradiated PLTs, initiating aggregation-induced TxA2 production. The experiments were also performed in aspirin (COX inhibitor) and imidazole (TxS inhibitor). Irradiation reduced aggregation by coagulation factor IV, indicating a decrease in sensitivity to TxA2 and inhibited COX and TxS, which are the major enzymes of the arachidonic acid pathway. These results contradict the data presented by Olban et al.^39^. In studies carried out on platelet-rich plasma (PRP) concentrates from pig blood. After irradiation with red laser light, the results showed an increased concentration of products reacting with thiobarbituric acid (TBARS). The TBARS assay detects the level of malondialdehyde (MDA), the primary lipid oxidation product. MDA is a COX product and a marker of arachidonate metabolism in PLT, but it can also result from non-enzymatic reactions. Even at the highest radiation doses, no outflow of lactate dehydrogenase from PLTs to the extracellular space was observed.

Since PBM inhibits PLT activation for all agonists, it most likely interferes with activating the coagulation system at a level common to all receptors^33^. Brill et al.^18^ postulated that the acceptor for R/NIR radiation responsible for changes in the coagulation system is located in PLTs performed first studies explaining this mechanism in vitro. During the experiments, individual blood fractions were irradiated in PRP and platelet-poor plasma (PPP) concentrates, PLTs subjected to gel filtration, erythrocyte concentrate, and leukocytes. The authors identified two chromophores that can absorb R/NIR radiation found in PLTs: guanylate cyclase (GC) and nitric oxide synthase (NOs) because both have heme. NOs additionally have flavin nucleotides as prosthetic groups. Activation of NOs leads to NO formation and stimulation of guanylate cyclase, which in turn causes cGMP to accumulate in the cell. The radiation increased the concentration of cGMP in the PLTs. The cGMP-dependent system regulates PLT activation, adhesion, and aggregation so that cGMP accumulation can inhibit them.

On the other hand, trials performed by Rola et al.^19^ revealed no significant changes in the PRMT-L-Arg/ADMA-DDAH axis resulting from the PBM, suggesting that it does not change the NO synthesis. Concomitantly they have not excluded NO as a factor responsible for reduced aggregation because it can be released from other blood cells (e.g. Hb-NO). Indeed, this hypothesis was partially confirmed by Wajih et al. recent research investigating far-red light PBM effects on PLT and a mixture of PLT and RBCs. They showed high efficacy and synergistic impact of PBM and nitrite treatment in PLT inhibition in the presence of RBCs. At the same time, the light mono-treatment was ineffective or insignificant when PRP or PLT and RBC were exposed, respectively. They concluded that the effect of PBM is strictly dependent on the NO amount bound to the RBCs in the process of previous nitrite bioactivation, so when light is applied alone, and NO amount is small, PLT activation is not inhibited (may even be stimulated). Otherwise, e.g. when the amount of NO is high, the activation of the PLTs is inhibited^36,37^.

Finally, R/NIR radiation causes biochemical changes in the membrane of PLTs in the lipid phase^35,40,41^. The authors suggest that the observed changes in the microviscosity of the lipid bilayer, also in the contact regions of lipids and proteins, may cause the activation of the cellular regulatory system and the synthesis of secondary messengers^40^. Such assumptions indicate that cell membrane structures contribute to the light modification of PLT activity. Despite clear evidence of the PBM anti-aggregation effects, the molecular mechanism of this action is not well understood. However, some explanation could be provided based on our studies on simpler models using liposomes and RBCs.

It is well established that PBM modifies cell membrane properties, decreasing its fluidity, reducing the polarity in the vicinity of the polar heads, and alternating the zeta potential^10,14^. Previously, we found no significant alterations of stored RBCs osmotic fragility after exposition to NIR PBM. However, the transitory part of the hemolysis curve was different, less dispersed, compared with the control one^23^. Due to mechanical stress-induced in vitro for the first time we could show that RBCs exposed to NIR PBM are more resistant to osmotic stress and less prone to hemolysis. It is in line with our previous animal studies where we observed lower PFHb, LDH, and serum bilirubin concentrations during extracorporeal circulation.

Moreover, quaternary transitions in Hb, dehydration of proteins of intact RBCs, and photodissociation of HbO_2_ both in vitro and in vivo result in the local O_2_ growth in the tissue due to exposition to NIR light were reported^42,43^. All the processes observed can be induced by the changes in water structure, which weaken the interactions between the water molecules and membrane surfaces and strengthen the hydrophobic effects. Finally, the build-up of the so-called exclusion zone is associated with the drop of pH inducing local acidification of the membrane environment^4^. The pH dependence of different agonists causing PLT aggregation reveals a lack of PLT activation at lowered pH^44, 45^, which could explain our observations assuming that the molecular mechanism of R/NIR light action is the effect of water structure changes.

It is worth highlighting that we designed and used the experimental setup to precisely control our studies’ PBM dose and sample temperature. None of the doses applied induced temperature changes. It is essential due to different methodological approaches used so far, including sample PBM at the room temperature or significant thermal effects observed due to laser action, far from physiological conditions. Obtained results may find clinical application in patients with hypercoagulation syndromes (as an alternative to drugs that block PLTs when intolerant) and when temporary and reversible inhibition of PLT function is desired. An example of the last case could be an extracorporeal circulation (ECC) which induces oxidative burst due to extravasated blood contact with large polymer surfaces. We recently demonstrated cytoprotective properties of blood PBM during 1 h ECC, which contributed to the limitation of PLT loss, inflammatory response, and RBC damage^17,20^.

### Limitations of the study

Extracellular vesicles (EVs) have attracted the interest of researchers in recent years because they play an important role in cell communication and the transport of diagnostically crucial chemicals. Because EVs are a heterogeneous set of membrane-covered nano and microparticles of varying sizes and shapes produced by various cell types, developing universally agreed techniques and interpreting data remains difficult^46^. The lack of ability to exclude the potential interference of significant PLT-EVs released under the impact of NIR light was a technical limitation of our investigation. Various procedures were used to assure the highest possible quality of PLT count (control of the size distribution, comparison of PLTs exposed to acetylsalicylic acid, which inhibits EV release^47^, with NIR PBM samples (Supplementary Materials)). So yet, no reports have been published on the release of PLT-EVs induced by NIR PBM. However, in a very recent article, Weihrauch et al. showed in bovine aortic endothelial cells that exposure to red light (670 nm) depletes intracellular S-nitroso protein while concomitantly increasing extracellular S-nitrosothiols embedded in extracellular vesicles (EV) of 200–400 nm, suggesting the mechanism of light-mediated vasodilation^48^. In summary, we cannot exclude the formation of large PLT-EVs as an effect of NIR PBM.

The study analysed the dose–response effects of NIR radiation on PLT count and function and damage to RBCs at the cellular level. Unfortunately, based on these data, we could not establish a direct link between the beneficial effects of PBM and the molecular mechanism that causes them. This has important implications for a proper understanding of the activation state of PLT after PBM. The reversible inhibition of PLTs observed in this study could be equivalent to the effect of PLT pre-activation. This process, which is also called priming, is defined as changes in the excitability threshold of PLT due to its modulation by bioactive molecules.

An example could be NO, which is considered a negative primer because its action raises the threshold for PLT activation^49^. The positive primer related to our model could be shear stress. Rahman et al. suggested that transient exposure to moderate or elevated upstream wall shear strain rates primed PLTs for significantly higher downstream adhesion and activation at the low shear strain rate^50,51^.

Interestingly, in our previous study where we applied the same NIR source for blood PBM during extracorporeal circulation using an artificial heart–lung machine in a porcine in vivo model, we observed elevated aggregation activity in the presence of agonists in the control group already at the 12th hour of observation. In contrast, PLT aggregation was never higher in the PBM group than preoperatively. So, keeping this in mind and the limitation of PLT loss and the pattern of CD62P expression, we concluded that NIR PBM might stabilize PLT function during CPB^20^.

Despite all the limitations, we provided a model that can further research NIR PBM interactions with blood cells and molecules, leading to better integration of physical science and cell biology.

## Conclusion

The methods used to date are far from necessary, so we have developed and employed a test setup to precisely regulate the dosage of PBM and the temperature of the blood sample. Here we showed under strictly reproducible in vitro conditions that NIR blood PBM with polychromatic light reversibly inhibits PLT activation in a dose-dependent manner. Discontinuation of the PBM leads to the cessation of the effect on PLT aggregation. Moreover, NIR PBM limits RBCs lysis.

## Supplementary Information

Supplementary Information.
