# Supplementary material for: Near-infrared photobiomodulation of blood reversibly inhibits platelet reactivity and reduces hemolysis
Source: Sci Rep. 2022 Mar 8;12:4042. doi: 10.1038/s41598-022-08053-y (PMC8904845; doi:10.1038/s41598-022-08053-y)
Supplement: Supplementary file 1 — Supplementary Information. [file 41598_2022_8053_MOESM1_ESM.docx]

**Supplementary information**

**Near-infrared photobiomodulation of blood reversibly inhibits platelet reactivity and reduces hemolysis**

Tomasz Walski^1^, Karolina Grzeszczuk-Kuć^1^, Katarzyna Gałecka^1^, Natalia Trochanowska-Pauk^1^, Raghvendra Bohara^2^, Albert Czerski^3^, Konstanty Szułdrzyński^4^, Wiesław Królikowski^5^, Jerzy Detyna^6^, Małgorzata Komorowska^1^

1. *Department of Biomedical Engineering, Faculty of Fundamental Problems of Technology, Wrocław University of Science and Technology, Wrocław, Poland*
2. *CÚRAM, SFI Research Centre for Medical Devices, National University of Ireland Galway, Ireland*
3. *Division of Pathophysiology, Department of Immunology, Pathophysiology and Veterinary Prevention, Wrocław University of Environmental and Life Sciences, Wrocław, Poland*
4. *Department of Anaesthesiology and Intensive Care, Central Clinical Hospital of the Ministry of Interior and Administration in Warsaw, Warsaw, Poland*
5. *2^nd^ Department of Medicine, Intensive Care Unit, Jagiellonian University, Medical College, Kraków, Poland*
6. *Department of Mechanics, Materials and Biomedical Engineering, Faculty of Mechanical Engineering, Wrocław University of Science and Technology, Wrocław, Poland*

**Corresponding author:**

**Tomasz Walski, PhD.**

Department of Biomedical Engineering, Faculty of Fundamental Problems of Technology, Wrocław University of Science and Technology, Wrocław, Poland;
e-mail: [tomasz.walski@pwr.edu.pl](mailto:tomasz.walski@pwr.edu.pl), Phone:+48713203168, Fax: +48713277727

**Contents**

**Supplementary distribution of the discoid platelet size**

**Supplementary Figures**

S1. The distribution of the PLT diameter counted with the Thrombo plus kit at different stages of the experiment.

S2. Changes in discoid shape PLT number during the experiment when aspirin (ASA) was added to the CTR or NIR (1.5 mW/cm^2^) sample after 0.5 h of incubation at 37 ℃.

S3. Changes in (A) the number of discoid shapes PLT count and (B) collagen-induced aggregation during the experiment were performed to compare the effect of aspirin (ASA) with the 1.5 mW/cm^2^ of NIR radiation.

**Supplementary Table**

Supplementary Table 1. The size of the discoid shape platelets counted with the Thrombo plus kit.

**Distribution of the discoid platelet size.**

The majority of the PLT-EVs are 100 – 300 nm in wavelength, which is clearly outside the detection range with the Thrombo plus kit and the method we used to detect nonactivated PLTs. Large PLT-EVs of 1 μm (discoid shape) or larger (nondiscoid shape) account for less than 0.5 percent of the total^1^. However, the significant number of EVs in plasma, in general, could potentially influence our data.

The PLT size of cells counted with the Thrombo plus kit (100 cells per group) was assessed at the preliminary stage of the experiment to eliminate potential interference from PLT-EV. Because of the interest in only nonactivated cells, discoid shape PLTs were measured at different time points. As shown in supplementary figure 1 and supplementary table 1 the diameter distribution of the counted cells was in the range of 1.9 to 3.7 μm.

Supplementary Table 1. The size of the discoid shape platelets counted with the Thrombo plus kit.

|  | CTR 0h | CTR 4h | CTR 8h | NIR 0h | NIR 4h | NIR 8h |
| --- | --- | --- | --- | --- | --- | --- |
| mean | 2.73 | 2.56 | 2.72 | 2.71 | 2.62 | 2.64 |
| SD | 0.28 | 0.29 | 0.38 | 0.30 | 0.28 | 0.35 |
| min | 2.07 | 1.92 | 2.02 | 1.99 | 1.94 | 1.95 |
| max | 3.43 | 3.24 | 3.60 | 3.62 | 3.34 | 3.64 |

Furthermore, additional experiments were performed to compare the effect of acetylsalicylic acid (ASA) on PLT with NIR PBM (irradiance of 1.5 mW/cm^2^). As shown in supplementary figures 2 and 3, there was no significant difference between these samples. ASA is a good PLT inhibitor^2^, and it has been reported to suppress PLT-EV release (like other antiplatelet drugs)^3^. Although both results, change in number and aggregation, were identical in both groups, we anticipate that the method we employed for discoid shape PLTs was appropriate and was not influenced by PLT-EVs. It should be noted that the ASA concentration used was relatively low (0.55 mmol/l) (equivalent to 10 mg/dl, normal non-toxic range 10-30 mg/dl), suggesting that the antiaggregatory impact could be even more substantial when a higher dose is employed.


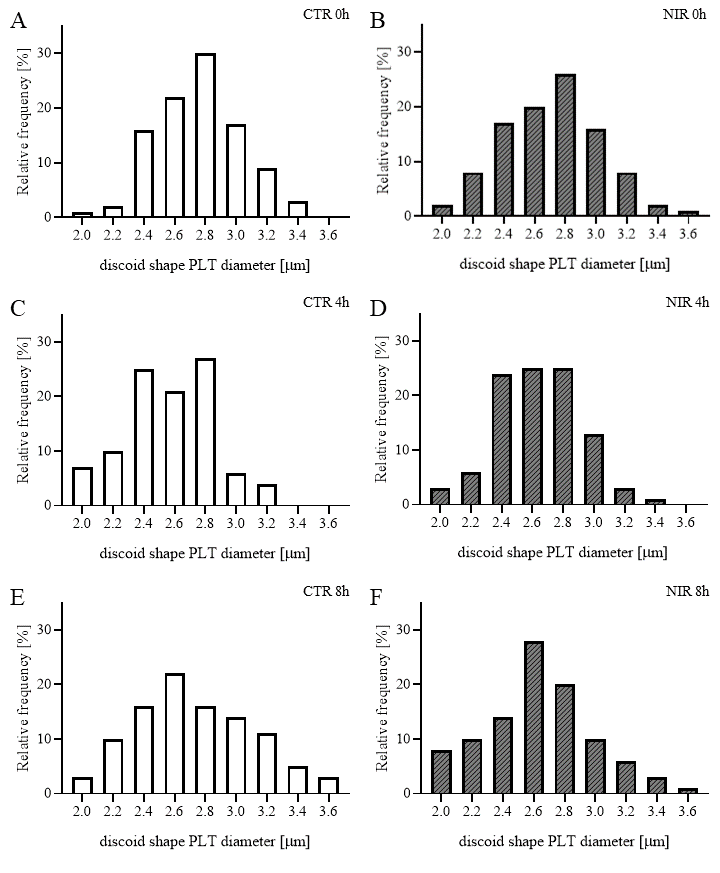


Supplementary Figure 1. The distribution of the PLT diameter was counted with the Thrombo plus kit at different stages of the experiment.


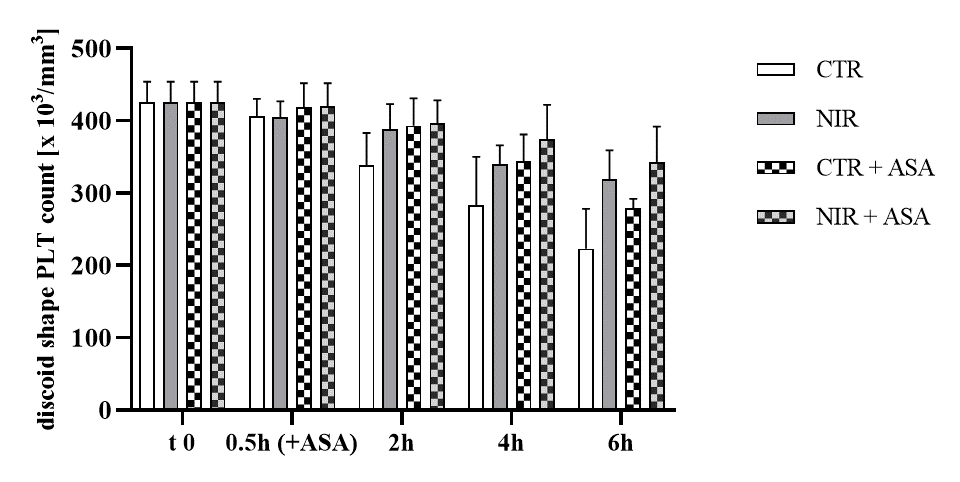


Supplementary Figure 2. Changes in discoid shape PLT number during the experiment when aspirin (ASA) was added to the CTR or NIR (1.5 mW/cm^2^) sample after 0.5 h of incubation at
37 ℃. The results are presented as the mean value and the standard deviation. The number of measurements n = 3.


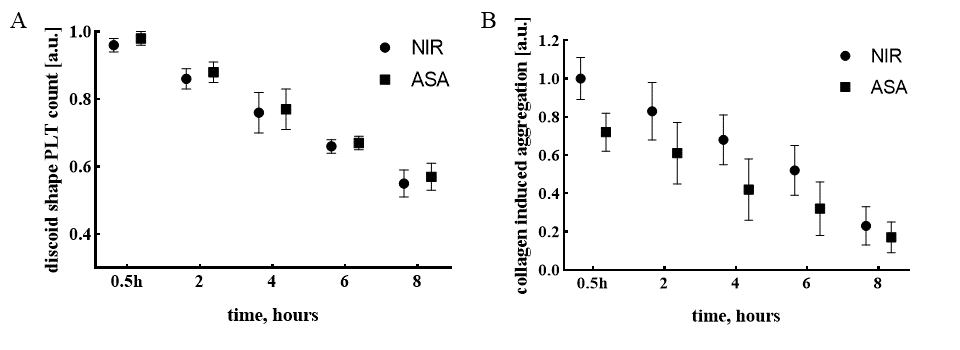


Supplementary Figure 3. Changes in (A) the number of discoid shapes PLT count and (B) collagen-induced aggregation during the experiment performed to compare the effect of aspirin (ASA) with the 1.5 mW/cm^2^ of NIR radiation. The results are presented as the mean value and the standard deviation. The number of measurements n = 8.

**Bibliography**

1. Arraud, N. et al. Extracellular vesicles from blood plasma: determination of their morphology, size, phenotype and concentration. J Thromb Haemost. **12**, 614-627 (2014).
2. Coppinger, J.A. et al. Moderation of the platelet releasate response by aspirin. Blood. **109,** 4786-4792 (2007).
3. Connor, D.E. et al. Effects of antiplatelet therapy on platelet extracellular vesicle release and procoagulant activity in health and in cardiovascular disease. Platelets. **27**, 805-811 (2016).
